# Fiscal Expenditure Structure, Vertical Fiscal Imbalance and Environmental Pollution

**DOI:** 10.3390/ijerph19138106

**Published:** 2022-07-01

**Authors:** Fengqin Qin

**Affiliations:** Chinese Academy of Fiscal Sciences, Beijing 100142, China; qinfengqin@chineseafs.org

**Keywords:** fiscal expenditure structure, expenditure on people’s welfare, vertical fiscal imbalance, environmental pollution

## Abstract

Based on China’s provincial panel data from 2007 to 2019, the authors of this paper conducted an empirical test on the direct effect of China’s fiscal expenditure structure on the reduction in environmental pollution with the use of a fixed effect model. We also creatively added an interaction item comprising vertical fiscal imbalance and the expenditure structure to further study the impact of vertical fiscal imbalance on reducing environmental pollution and its effect on the fiscal expenditure structure. The study results show that a structure in favor of expenditure on people’s welfare noticeably reduces environmental pollution. However, after the introduction of the vertical fiscal imbalance indicator, the pollution reduction effect decreases. That is, the vertical fiscal imbalance weakens and distorts the impact of the fiscal expenditure structure on the reduction in environmental pollution. Therefore, it is possible to further motivate local governments with incentive measures, such as fiscal decentralization and the centralization of administrative responsibilities, and regulate the environmental pollution of local governments through use of restrictive measures, such as the “green GDP” evaluation mechanism to further improve the fiscal expenditure structure of local governments, enhance the environmental pollution reduction capability of fiscal expenditure.

## 1. Introduction

With the rapid development of the economy and society, problems such as climate change, biodiversity decline or environmental pollution are getting worse, and global environmental remediation faces challenges. Environmental pollution is a long-running problem that has occurred alongside industrialization and is the byproduct of the industrialization of all the countries in the world. Although many countries have taken powerful action for environmental remediation, the government is not the only body to make improvements. Market entities and the public are also integral parts. Tang and Mazmanian deemed that policy cannot be implemented by solely relying on the public sectors or a single organization. Policy implementation needs the participation of the public sectors, private sectors, and the public [1]. Shen and Liu proposed a “government–society–market” collaborative improvement model [2].

The environmental problem is not only the problem of production structure, international trade, and finance interdependence. Wang and Liu et al., studied China’s industrial structure and deemed that it is closely related to the environment and that technical progress would greatly promote improvement in environmental quality [3,4]. Regarding the research on international trade problems, Xu et al., and Levinson have mainly conducted studies on the impact of American import and export products on environmental pollution and consistently found that environmental pollution would be reduced by importing products of less intensive pollution [5,6]. As for the financial problem, Hanh et al., studied the impact of foreign direct investment on environmental pollution [7]. Zioto et al., deemed that finance interdependence would have a positive effect on the environment [8]. In addition, it is also an education problem. Armstrong et al., and Aguilar-Jurado et al., while conducting research on the problem of education and the environment, also found that it is possible to enhance environmental awareness by adding resource recovery courses to education curriculums [9,10]. Moreover, the environmental problem is a problem of the position from which we perceive reality.

Similar to most industrialized countries, China has taken the road of “pollution first, governance later”. Events such as water pollution in Taihu Lake in 2007 and smog in Beijing show that China’s environmental pollution exceeds its load-carrying capacity. The severe conditions and the difficulty of remediation have been fully shown. For this reason, as the body that provides public goods, such as the environment, and that controls environmental pollution, the government conducts environmental remediation by fully making use of policy tools, such as fiscal policy, so as to continuously enhance environmental quality. During this process, different expenditure structures show different impacts on the reduction in environmental pollution. According to China’s expenditure function classification, we divided the fiscal expenditure into people’s welfare and productive expenditure, which can also be sorted by the degree of pollution. Environmental protection, education, science and technology, etc., which are included in the expenditure on people’s welfare, contribute almost no pollution. Lopez et al., also deemed that expenditures in terms of education, medical care, environmental protection, science and technology, etc., have strong positive externality, whereas transportation, resource exploration, etc., included in the productive expenditure category, may cause pollution to some extent, which means they have negative externality [11].

There are certainly many factors that affect environmental remediation. In addition to the international and domestic vertical cooperation or interaction of concern to us all, social consciousness also cannot be ignored. While conducting a study on the impact of environmental awareness of city consumers on public policy, Wang et al., found that social responsibility consciousness and ecological environmental protection consciousness have direct and indirect impacts on public decisions [12]. Sudibyo and Sutanto found in their research that social responsibility consciousness and the environmental protection consciousness of enterprises positively affect the green capital [13]. Vertical fiscal imbalance is one of the most significant influential factors. Therefore, the main research goal of this study was to investigate the impact of vertical fiscal imbalance on the environmental pollution remediation effect of the fiscal expenditure structure.

China’s tax-sharing system reform was implemented in 1994. After several reforms, the fiscal system formed has the characteristics of “responsibility decentralization” and “finance centralization”. The financial power of the central government is greater than its administrative responsibilities, whereas the financial power of the local government is not enough to match its administrative responsibilities, which is a common vertical fiscal imbalance phenomenon in decentralized countries. Moreover, as local governments have more decision-making power for economic affairs, the level of discrepancy between the revenue and expenditure of local governments rises as well, and the vertical fiscal imbalance problem becomes worse. Although the fiscal imbalance phenomenon is inevitable, excessive fiscal imbalance is bound to cause severe economic, societal, and governance problems. For example, the vertical fiscal imbalance level affects the environmental pollution reduction effect of the fiscal expenditure structure. The aim of this study is to investigate the problem that vertical fiscal imbalance weakens and distorts the pollution reduction effect of the local government fiscal expenditure structure.

The rest of this paper is organized as follows: Section 2 presents a literature review and the research hypothesis. Section 3 presents the empirical design. Section 4 presents an empirical analysis. Section 5 includes the research conclusions and policy implications.

## 2. Literature Review and Research Hypothesis

### 2.1. Impact of Fiscal Expenditure Structure on Environmental Pollution

Since the occurrence of environmental pollution, the possible impact of the government has been the research focus of scholars. Public finance is not only an important economic tool of the government but also the base and mainstay of state governance. Therefore, it plays an important role in environmental pollution remediation. As an important part of public finance, fiscal expenditure is indispensable in environmental pollution remediation. The environmental effect of the fiscal expenditure structure is also a focus of research. Some scholars have explored the impact mechanism from the angle of the fiscal expenditure structure. Wu et al., found that the appeal for political achievements urges local officials to allocate limited resources to areas conducive to economic growth, thereby reducing financial expenditures in people’s welfare areas such as environmental protection, which is harmful to efforts to improve environmental quality [14]. Halkos et al., utilized the data of seventy-seven countries in the world from 1980 to 2000, conducted an empirical study on the direct effect of fiscal expenditure on environmental pollution, and found that expenditure in favor of economic growth is bad for environment quality improvement [15]. He et al. Found that the expenditure structure in favor of public services, such as environmental protection, and education, is conducive to reducing environmental pollution [16]. Halkos et al., identified four affecting mechanisms of the effect of fiscal expenditure on the environment by establishing theoretical and empirical models and found that fiscal expenditure in favor of providing public goods is beneficial to reducing environmental pollution in production [17]. Chen et al., conducted a more detailed classification of the effect of fiscal expenditure on the environment, including the technique effect, consumer preference effect, economic scale effect, factor substitution effect, and revenue control effect, and found that increasing the share of non-economic fiscal expenditure is conducive to alleviating production-generated pollution [18]. Yu et al., held that by the means of technology, education, and so on, non-economic public expenditure would promote the technological innovation of emission reduction and pollution remediation, and thus the environmental quality could be improved at the same time as reducing pollutant emissions [19]. Lu et al., further found that increasing the share of China’s non-economic fiscal expenditure is conducive to alleviating the regional emission levels of consumption-generated pollutants [20].

### 2.2. Mechanism of the Effect of Vertical Fiscal Imbalance on Environmental Pollution

A study on the practice of fiscal decentralization conducted by Bordignon et al., indicated that, in reality, fiscal decentralization fails to completely achieve institutional balance. Instead, since financial power is controlled by the central government, the central government has more revenue and less expenditure, whereas the local governments have less revenue and more expenditure. The fiscal surplus of the central government and the revenue shortfall of the local government may inevitably lead to vertical fiscal imbalance [21]. Many scholars, such as Boadway and Jiménez-Rubio, believe that vertical fiscal imbalance is a common phenomenon in all decentralized countries, and an appropriate vertical fiscal imbalance would promote the improvement of the local public expenditure structure [22,23,24]. However, Bradman et al., deemed that although vertical fiscal imbalance is commonly found in decentralized countries, excessive imbalance may have a negative effect and cause enormous harm. For example, the fiscal decentralization of developing countries would cause disjunction between their revenue and expenditure, thereby distorting the public expenditure structure of the local governments [25]. Smith et al., analyzed the political decentralization and fiscal decentralization of six countries in Latin America, including Argentina and Mexico, and found that excessive power of the provinces would cause severe vertical fiscal imbalance [26]. Chu et al., found that China’s fiscal decentralization system is different from that of the fiscal federalism countries in the West that simultaneously promote economic decentralization and political decentralization; China’s decentralization system, with political centralization and economic decentralization, leads to more serious vertical fiscal imbalance [27]. Duan et al., deemed that fiscal transfer payments among governments are an important measure for easing vertical fiscal imbalance [28]. In a study, Boett et al., found that the Italian government’s enhancement of the degree of self-financing of lower-level governments was conducive to easing vertical fiscal imbalance [29]. In a study on South Africa, Amusa et al., found that enhancing the degree of local self-sufficiency was more beneficial to easing vertical fiscal imbalance, instead of fiscal transfer payments among governments [30]. Oates believed that excessive vertical fiscal imbalance may make local governments rely too heavily on transfer payments. Local governments’ efforts would be decreased under the soft constraint, thereby damaging the financial autonomy of the local government [31]. On the basis of comprehensively measuring China’s vertical fiscal imbalance level, Chu et al., further studied its impact on public expenditure structure bias and found that vertical fiscal imbalance affects the public expenditure behavior of the local government and causes distortion of the public expenditure structure [32]. Jia et al., investigated the impact of vertical fiscal imbalance and political promotion on land finance and found that vertical fiscal imbalance exacerbates the behavior of land finance, during which political promotion intensifies the behavior, which affects the sustainable development of China’s economy and society [33].

Although there have already been some studies related to the impact of fiscal expenditure structure bias on the environment, the factors affecting the expenditure structure have not received much attention. Wang et al., studied the relationship between vertical fiscal imbalance and environmental pollution. When faced with vertical fiscal imbalance constraints, especially a high level of imbalance, local governments depend on tax revenue from polluting enterprises to ease pressure, which inevitably causes environmental pollution [34]. Xi and Xi et al., discovered that when faced with vertical fiscal imbalance, governments use fiscal incentives to alleviate the imbalance. For example, fiscal expenditure may be biased toward industries that can bring more fiscal revenue, such as the construction industry and real estate industry, but these industries also cause environmental pollution. Under the influence of vertical fiscal imbalance, with respect to the fiscal expenditure structure, governments may lean more towards “resource-intensive” projects, represented by infrastructure construction. Such expenditure increases the GDP in a short time but causes irreversible pollution [35,36]. Vertical fiscal imbalance does not directly pollute the environment, but it affects the decision behavior of local governments. Local governments may seek economic development projects that can take effect in a short term in order to ensure the stable growth of fiscal revenue. Furthermore, local governments prioritize fiscal expenditure on investments that can rapidly bring in revenue. Public goods related to people’s welfare that take a long time to gain revenue, such as environmental protection, are becoming more inefficient, which inevitably affects the impact of the fiscal expenditure structure on pollution. The following Table 1 shows the main characteristics of the most important articles of the literature.

### 2.3. Research Hypotheses of This Study

It can be seen from the above literature review that the existing studies have explored the fiscal expenditure structure and vertical fiscal imbalance in terms of their impact on environmental pollution. Studies on the combined impact of these two and their interaction on reducing environmental pollution have rarely been conducted. If the effect of one aspect is ignored, besides causing possible problems in terms of assessment results, it is possible to ignore the affecting mechanisms that have not been noticed before, for example, whether vertical fiscal imbalance affects the impact of the fiscal expenditure structure on pollution reduction. Ignoring these problems would probably skew our perceptions and even mislead decision-making in some cases.

The aim of this study was to investigate the combination of expenditure on people’s welfare and vertical fiscal imbalance, which has not been examined by prior research and is also the innovation of this paper. The impact of expenditure on people’s welfare and vertical fiscal imbalance on reducing environmental pollution was selected largely for the following two reasons: First, the Chinese government has always adhered to people-oriented governance and views the people’s welfare as one of the two essential tasks of social construction. The main contradiction in Chinese society at the present stage is a conflict between the people’s growing demand for a better life and the unbalanced and inadequate development. Public finance, as the foundation and mainstay of state governance, plays an irreplaceable role in environmental regulation. Correspondingly, the proportion of expenditure on people’s welfare in the local government’s public expenditure may indicate whether the people’s welfare provision function is achieved or not. Furthermore, the environment, as a public good, is also an aspect of expenditure on people’s welfare. Productive expenditure corresponds to expenditure on people’s welfare. We mainly studied expenditure on people’s welfare, in which environmental expenditure is included. According to the study of Lu et al., as well as Devarajan et al., the sum of expenditures, including general public service, public safety, science and technology, social security, education, culture, sports, and media, as well as energy conservation and environmental protection, was viewed as expenditure on people’s welfare [20,37]. Second, vertical imbalance, as a characteristic of a decentralized country, is evident in China. In prior studies, most researchers have paid attention to the impact of vertical fiscal imbalance on the public expenditure structure or how vertical fiscal imbalance affects environmental pollution. For example, Li et al., found that vertical fiscal imbalance causes environmental degradation by changing the industry structure, and also restrains environment supervision [38]. Huang et al., found that in China, a higher level of vertical fiscal imbalance causes even worse environmental pollution [39]. In summary, we found that prior studies have ignored the impact of vertical fiscal imbalance on the environmental pollution reduction effect of the fiscal expenditure structure. Therefore, this study combined vertical fiscal imbalance with expenditure on people’s welfare to comprise an interaction item to further investigate. This interaction is the innovation of this study and of great importance. On these grounds, we propose the following hypotheses:

**Hypothesis** **1**: *Expenditure on people’s welfare is conducive to reducing the emissions of environmental pollutants, thereby enhancing environmental quality*.

**Hypothesis** **2**: *Vertical fiscal imbalance may decrease the environmental pollution reduction effect of expenditure on people’s welfare.*

## 3. Empirical Design

### 3.1. Model Building

According to the hypotheses mentioned in the above text, this paper mainly explores the mechanism for the fiscal expenditure structure to affect the environmental pollution. The fixed effects method is used, mainly for the reason that the panel data have a large quantity of strata, which may control the unobservable individual heterogeneity and solve the omitted variable bias at a certain extent. A result *p*-value = 0.001 < 0.5 is obtained from the Hausman test [17]. A fixed effect model is more suitable for the sample of this research.

(1) $${\mathrm{Pollut}}_{\mathrm{it}}=\beta_{0}+\beta_{1}\exp_{\mathrm{it}}+\beta_{2}X_{\mathrm{it}}+\mu_{i}+\lambda_{t}+\varepsilon_{\mathrm{it}}$$

Since the impact of the fiscal expenditure structure on environmental pollution reduction is affected by vertical fiscal imbalance (VFI), we added the vertical fiscal imbalance indicator into Model (1); the specific model is shown as follows:

(2) $${\mathrm{Pollut}}_{\mathrm{it}}=\beta_{0}+\beta_{1}{\mathrm{vfi}}_{\mathrm{it}}+\beta_{2}\exp_{\mathrm{it}}+\beta_{3}{\mathrm{vfi}}_{\mathrm{it}}\times \exp_{\mathrm{it}}+\beta_{4}X_{\mathrm{it}}+\mu_{i}+\lambda_{t}+\varepsilon_{\mathrm{it}}$$

where the explained variable Pollut_it_ denotes No. i province’s industrial pollution in the year t. This study adopted emissions per capita of wastewater (pww), waste gas (pwg), and solid waste (psw) as the explained variables, and processed the explained variables using a logarithm. exp_it_ denotes the core explanatory variable, i.e., the fiscal expenditure structure. vfi_it_ denotes the moderator variable, i.e., vertical fiscal imbalance. X denotes a control variable set. $\mu_{i}$ and $\lambda_{t}$ denote the region and time fixed effect, respectively. $\varepsilon_{\mathrm{it}}$ denotes a random error item.

### 3.2. Variable Descriptions

#### 3.2.1. Core Explanatory Variables

There are two main calculation methods for the fiscal expenditure structure, one of which is represented using the proportions of various fiscal expenditures of the government’s total fiscal expenditure (Wang et al., Yu et al.) [19,40]. The other is reflected by ratios of the amounts of various fiscal expenditures and different combinations thereof (Tong) [41], but the first method is usually adopted. Expenditure on people’s welfare is the most direct carrier of the people’s welfare provision function of the local government. Therefore, this study adopted the proportion of expenditure on people’s welfare in which the environmental protection expenditure is included in the government’s total fiscal expenditure to represent the fiscal expenditure structure. The specific computing formula is shown as follows:

(3) $$\exp=\frac{\mathrm{PWE}}{{\mathrm{LG}}_{s}}$$

where exp denotes the fiscal expenditure structure. A greater value of exp indicates that the fiscal expenditure structure is greatly in favor of the expenditure on people’s welfare. In contrast, a smaller value of exp indicates the fiscal expenditure structure is greatly in favor of economic expenditure. PWE denotes the expenditure on people’s welfare, and LG_s_ denotes the local government’s public expenditure.

#### 3.2.2. Moderator Variables

In a multi-level government system, governments at all levels can afford their fiscal expenditure with their own revenue and never have to rely on the transfer payment system between the superior and subordinate governments, which means that the government at all levels is in an ideal fiscal balance state. Thus, the opposite indicates a vertical fiscal imbalance state. There are many measures related to vertical fiscal imbalance. Since Chinese decentralization has typical asymmetry due to political centralization, based on the research of Eyraud and Lusinyan [42], this study conducted measurements according to the formula of Chu et al. The specific formula is: VFI = 1 – (revenue decentralization/expenditure decentralization) × (1 − local government’s gap ratio of fiscal self-sufficiency), where revenue decentralization and expenditure decentralization are based on the measurements of Chu and Zhao [43]. When expenditure decentralization increases and revenue decentralization decreases, the Chinese vertical fiscal imbalance increases even more.

(4) $$\mathrm{VFI}=1-\frac{{\mathrm{FD}}_{r}}{{\mathrm{FD}}_{s}}\times \left(1-\mathrm{LBD}\right)$$

(5) $${\mathrm{FD}}_{r}=\frac{{\mathrm{LG}}_{r}/\mathrm{Lpop}}{\left({\mathrm{LG}}_{r}/\mathrm{Lpop}\right)+\left({\mathrm{CG}}_{r}/\mathrm{pop}\right)}$$

(6) $${\mathrm{FD}}_{s}=\frac{{\mathrm{LG}}_{s}/\mathrm{Lpop}}{\left({\mathrm{LG}}_{s}/\mathrm{Lpop}\right)+\left({\mathrm{CG}}_{s}/\mathrm{pop}\right)}$$

(7) $$\mathrm{LBD}=\frac{{\mathrm{LG}}_{s}-{\mathrm{LG}}_{r}}{{\mathrm{LG}}_{s}}$$

where VFI denotes vertical fiscal imbalance; FD_r_ denotes fiscal revenue decentralization; FD_S_ denotes fiscal expenditure decentralization; LG_r_ and LG_s_ denote public revenue and expenditure of the local government, respectively; CG_r_ and CG_s_ denote public revenue and expenditure of the central government, respectively; Lpop and pop denote the total population of the locality and the whole country, respectively; LBD denotes the local government’s gap ratio of fiscal self-sufficiency.

According to the computing formula related to the vertical fiscal imbalance level in the above text, we determined the vertical fiscal imbalance level of 31 provinces from 2007 to 2019, and the results show the following:

China has a higher level of vertical fiscal imbalance. There are two causes for this phenomenon, one of which is China’s political centralization system. That is, China’s fiscal decentralization is carried out under the centralized administration system [44]. Although China started to implement tax-sharing system reform in 1994, it is greatly different from the decentralization system of federalism in the West. Unlike federal governments in the West, China does not give a high level of autonomy to the local governments. The central government has absolute authority in the administration system, thus under the China-specific system, with political centralization and economic decentralization, the allocation of central and local public finance means that more fiscal revenue is turned over and more expenditure and administrative responsibilities are transferred to the lower-level government (Bordignon and Du) [21,45]. Another reason is the historical background of the emergence of China’s tax-sharing system. In order to strengthen the financial resources of the central government and enhance macro-economic control capability, China started to implement tax-sharing system reform and effectively turned around the unfavorable situation of the continuous decline of the revenue and expenditure of central public finance by partitioning tax categories and setting up a shared tax. However, another problem occurred at the same time this problem was solved. The central government has absolute authority in fiscal allocation. The expenditure and administrative responsibilities of the local governments have not changed, which inevitably causes the problem of vertical fiscal imbalance (Chu et al.) [32].

Over time, China’s vertical fiscal imbalance has improved to a certain extent, but the speed of improvement has been relatively slow. Although China has a high level of vertical fiscal imbalance, changes have been minimal. The reason for the emergence of this phenomenon is China’s highly centralized political system, the constraints of which cause China’s vertical fiscal imbalance to be worse than that of the fiscal federalism countries in the West. Starting with higher levels of imbalance can transfer the incentives of the central government to the local government, further affecting the operating efficiency of the local government, which would have a negative effect. The Chinese government also arranged relevant transitional measures at the beginning of the implementation of the tax-sharing system, appropriately expecting an increase in the vertical fiscal imbalance. These measures were meant to avoid the emergence of this situation to a certain extent. However, the state adjusted individual and the local business income tax from a local to a shared tax in 2001 and determined the ratio of the shared tax between the central and the local governments as 6:4 after 2002. The tax sharing proportion of the central government was further enhanced, causing the financial power of the central government to be larger than its administrative responsibility. Thus, the discrepancy between the local government’s administrative responsibility and its expenditure responsibility has increased.

#### 3.2.3. Control Variables

In order to avoid variable omissions causing estimation bias, this study adopted foreign direct investment (fdi), energy consumption structure (ecs), industrial structure (is), urbanization rate (urba), real GDP per capita (rgdp), population density (pd), fixed asset investment (fai), and built-up area (bua) as control variables. Descriptions of the main variables are shown in Table 2.

### 3.3. Data Sources and Descriptive Statistics

In consideration of the availability of the data and completeness of the sample, this study mainly explored samples from 31 provinces, autonomous regions, and municipalities of mainland China (excluding Hong Kong, Macao, and Taiwan) from the time period of 2007–2019. Since expenditure on people’s welfare includes environmental protection expenditure, the latter is classified as governmental revenue and expenditure, so the sample period started in 2007. The economic data and environmental pollution data were obtained from the Finance Yearbook of China, China Statistical Yearbook on Environment, China Statistical Yearbook, China Industry Economy Statistical Yearbook, the website of the National Bureau of Statistics (National Bureau of Statistics: http://www.stats.gov.cn/ (accessed on 10 April 2022)), the website of the Ministry of Ecology and Environment of the People’s Republic of China, (Ministry of ecological and environment of the people’s Republic of China: https://www.mee.gov.cn/ (accessed on 10 April 2022) and the CEI Statistics Database (https://data.cnki.net/HomeNew/index (accessed on 10 April 2022)). The descriptive statistical results of the main variables are shown in Table 3.

## 4. Empirical Analysis

### 4.1. Basic Regression

Table 4 reports the basic regression results. Columns (1), (3), and (5) correspond to Hypothesis 1, whereas columns (2), (4), and (6) correspond to Hypothesis 2. According to the results in columns (3) and (5), there are significant negative relationships among the fiscal expenditure structure, per capita industrial waste gas, and per capita solid waste. Column (1) is not included due to insignificant results. Furthermore, the coefficient is significant at the statistical level of 1%, which indicates that expenditure on people’s welfare, in which the environmental protection expenditure is included, has an obvious effect on environmental pollution reduction. This is consistent with both Hypothesis 1, mentioned in the text above, and the research conclusions of Halkos and Paizanos [17]. However, the expenditure structure is affected by other factors. Excessive vertical fiscal imbalance is one of the factors affecting the fiscal expenditure structure. In order to study the moderating effect of vertical fiscal imbalance on the pollution reduction effect of the expenditure structure, we added the interaction items of the expenditure structure and vertical fiscal imbalance into the research, as shown in columns (2) and (6). It was found that after the addition of vertical imbalance, the effect of expenditure on people’s welfare on pollution reduction decreased, which is not conducive to improving environmental quality. This also verifies the validity of Hypothesis 2. It can be seen in Table 4 that for other control variables, the coefficients of the industrial structure and energy consumption structure are positive, which indicates that both industrialized development and the typical energy consumption structure are significant factors that cause environmental pollution. At the early stage, China’s environmental pollution came from industrial development.

### 4.2. Tackling Endogeneity Issues

Due to various reasons, including simultaneity, unobservable heterogeneity, dynamic heterogeneity, etc., there may be endogenous problems, so certain methods should be used to solve them (Arvanitis, Stamatopoulos, Terzakis) [46]. A generalized method of moments (GMM) can effectively solve endogenous problems (Arellano and Bond; Arellano and Bover; Blundell and Bond; Roodman) [47,48,49,50]. It can be seen by comparing Table 4 above with Table 5 below that the regression coefficients of the core explanatory variable remain largely unchanged, with some adjustments to the magnitude and significance of the coefficient values. The situations of the other control variables are basically in accordance with those in Table 4. As space is limited, they are not reported here.

### 4.3. Robustness Tests

Given that a change in the indicator may affect the results of the empirical analysis, in order to verify its robustness, we replaced the indicators of the explained and moderator variables to conduct a robustness test on the results of the empirical analysis of this study.

#### 4.3.1. Environmental Pollution Indicator Change (Total Amount of Three Types of Industrial Waste)

In the basic regression, we used three types of waste per capita as the indicators of the amount of discharged pollutants, but the total discharge is also a commonly used measurement indicator. Therefore, Table 6 reports the results of the aggregate relation between the fiscal expenditure structure and three types of industrial waste, under the effect of vertical fiscal imbalance. As shown in the results, the fiscal expenditure structure has a significant negative effect on environmental pollution. Furthermore, the coefficients of wastewater and solid waste are significant at the statistical levels of 1% and 5 %, which indicates that the sum of education expenditure, health expenditure, science and technology expenditure, environmental expenditure, and so on, would have a positive effect on the environment. That is to say, with an increase in the expenditure on people’s welfare, the pollution reduction effect would improve. It can be seen from column (6) that the coefficient of the interaction item of expenditure on people’s welfare and vertical fiscal imbalance is significant at a statistical level of 1%. Therefore, it can be concluded that the higher the vertical fiscal imbalance level is, the lower the reduction effect of expenditure on people’s welfare is. This is basically consistent with the basic regression results, which indicates their robustness.

#### 4.3.2. Environmental Pollution Indicator Change (Emission Intensity of Three Types of Industrial Waste)

Since industrialization is the main force behind the rapid growth of the amount of environmental pollution emissions caused by the industrial added value per unit (i.e., pollution intensity), which is able to reflect the environmental efficiency of China’s industrialization, we adopted the pollution intensity indicator to measure China’s industrial pollution level from a quality perspective. The specific results are shown in Table 7. It can be seen that the regression results are relatively consistent with the basic regression results. The coefficient of column (1) is significant at a statistical level of 1%. After the addition of the interaction item, it can also be seen in column (2) that its results are not significant. That is to say, the pollution reduction effect of the fiscal expenditure structure is greatly decreased. The expenditure and interaction item of column (6) is significant at the statistical level of 1%, which indicates that vertical fiscal imbalance weakens the pollution reduction effect of expenditure on people’s welfare. This is consistent with the basic regression results, verifies our hypotheses, and proves the robustness of the basic regression results.

#### 4.3.3. Vertical Fiscal Imbalance Indicator Change (Fiscal Pressure)

The two robustness tests above were both performed by changing the explained variables. However, as the main expressive form of vertical fiscal imbalance, fiscal pressure can be used instead of vertical fiscal imbalance to conduct a robustness test. The fiscal pressure in this study represents the gap between fiscal revenue and fiscal expenditure (fiscal pressure = fiscal expenditure-fiscal revenue). Table 8 denotes the results of regressing the environmental pollution reduction effect of fiscal pressure. As shown in the results, the effect of the expenditure structure on the three types of waste per capita has significant negative relationships, and the main coefficients are significant at the statistical level of 1%, which indicates that the expenditure structure, presented by the expenditure on people’s welfare, has a reduction effect upon environmental pollution. This is consistent with the above results, which also fully indicates that Hypothesis 1 is supported. After the addition of fiscal pressure, the interaction item of the expenditure structure and fiscal pressure is still significant, which indicates that fiscal pressure would affect the pollution reduction effect of the expenditure on people’s welfare. That is, the heavier the fiscal pressure is, the lower the reduction effect is. This result shows that Hypothesis 2 is reasonable. The other regression results are basically consistent with the above results, which indicates the robustness of the basic regression results and verifies the basic hypotheses.

### 4.4. Regional Heterogeneity Analysis

China can be divided into eastern, central, and western regions according to several factors, including geographical position and economic development level. There are significant differences in the fiscal expenditure structure and vertical fiscal imbalance levels among different regions. Thus, in order to conduct better research on the effect of the vertical fiscal imbalance of different regions on the pollution reduction effect of expenditure structure, we conducted the heterogeneity testing separately on the eastern, central, and western regions.

Table 9 displays the results of the reduction effect of expenditure on people’s welfare after the interaction item of vertical fiscal imbalance was added to the expenditure on people’s welfare of the eastern, central, and western regions. As shown in the table, the regression results by region have obvious differences and produce different effects on different pollutants. The regression results of the eastern region merely affect wastewater to a certain extent, and its coefficient is significant at the statistical level of 1%. The regression results of the central region are relatively more obvious, and its coefficient is significant at the statistical level of 5%. Under the effect of vertical fiscal imbalance, the reduction effect of the expenditure on people’s welfare decreases. There is a significant negative relationship between the people’s welfare fiscal expenditure structure of the western region and the amount of discharge of the three types of waste, and the coefficient is significant at the statistical level of 1% or 5%. This indicates that after the addition of the vertical fiscal imbalance interaction item, the pollution reduction effect of expenditure on people’s welfare decreases.

The main reason for the emergence of these differences among the eastern, central, and western regions is the lower economic development level of the western region. The ecological environment of most regions is under key protection, so the addition of fiscal expenditure is more favorable to environmental protection expenditure, which would inhibit environmental pollution. For the sake of economic development, the fiscal expenditure of the central region would be more favorable to productive expenditure, which can enhance the economic development level and increase fiscal revenue in a short time, exacerbating environmental pollution. The other control variables are consistent with the basic regression results, and unnecessary details are not given here. The results of the eastern region are not significant, because the economic development level of the eastern region is relatively high, and the vertical fiscal imbalance level is low. In addition, its fiscal expenditure structure is relatively reasonable, which produces minor effects. This is consistent with our hypothesis and the basic regression results. Hypotheses 1 and 2 and the robustness of the basic regression results were further proved by the heterogeneity test. This also shows that different regions may need to implement different fiscal response policies.

## 5. Conclusions and Policy Recommendations

### 5.1. Research Conclusions

First, the impact of the fiscal expenditure structure directly on environmental pollution was analyzed by constructing a panel data model. The fiscal expenditure structure here was measured as the ratio of the expenditure on people’s welfare (which includes the general public service expenditure, public safety expenditure, science and technology expenditure, social security expenditure, education expenditure, culture, sports, and media expenditure, as well as energy conservation and environmental protection expenditure) in relation to the total fiscal expenditure of the local government. This research found that expenditure on people’s welfare plays a role in reducing environmental pollution.

Second, under the impact of vertical fiscal imbalance, the effect of the fiscal expenditure structure on reducing environmental pollution decreases. We introduced the vertical fiscal imbalance indicator as a moderator variable, constructed an interaction item with the fiscal expenditure structure, and further studied its impact on environmental pollution. It was found that vertical fiscal imbalance weakens or distorts the environmental pollution reduction effect of the local government’s expenditure structure.

### 5.2. Policy Recommendations

As part of the conclusion of this paper, we propose recommendations regarding incentives and constraints for the next stage of China’s environmental pollution remediation.

#### 5.2.1. Incentive Measures

First, part of the financial power should be appropriately transferred to lower levels of government. In order to solve the vertical fiscal imbalance problem, which has existed for a long time and is possibly becoming worse, appropriately transferring part of the financial power to lower levels of government should be taken into consideration. For example, it is possible to appropriately enhance the proportion of shared tax in the lower levels of government so that local governments are able to obtain tax revenue commensurate with their fiscal expenditure from economic growth. In addition, some stable main local taxes can be cultivated. Thus, when confronted with decreases in tax shares, the local government may be less pressured when dealing with large fiscal expenditures. It is also advised to find more methods of increasing tax revenue and non-tax revenue, which can better relieve the pressure caused by vertical fiscal imbalance.

Second, part of the local fiscal administrative responsibility should be transferred to a higher level of government and the division of the administrative and expenditure responsibilities between governments should be improved. According to the Division Plan of Administrative Responsibility between the Central and Local Governments in the Field of Ecological Environment (issued by the General Office of the State Council, 13th in 2020), it is possible to relieve the expenditure pressure on the local governments to the greatest extent by appropriately enhancing the administrative responsibility of the central government in terms of cross-regional ecological environmental protection, improving the division of the administrative and expenditure responsibilities between the governments, and providing a certain incentive for the local governments to successfully implement heavier environmental regulation. Moreover, the government with greater administrative responsibility should consider the relationship between the market and government. By providing more guidance for society and enhancing society’s enthusiasm for taking part in environmental remediation, the problem then can be solved more efficiently and thoroughly.

#### 5.2.2. Constraint Measures

First, “GDP-oriented” evaluation should gradually become a “green GDP-oriented” evaluation. When confronted with fiscal pressure or a vertical fiscal imbalance problem, the local government can not only choose to take the “demutualization” action but can also be encouraged to develop “green GDP” for implementing the evaluation, which can be considered a means of promotion of local government officials. In this way, it is possible to motivate the local government to recognize the importance of green development and enlarge the scale of fiscal expenditure on people’s welfare, such as energy conservation and environmental protection. Making the government’s behavior more public can provide a better environment for the public, and this practice can also adapt to China’s main contradictions at the present stage. The local governments undertake a tremendous amount of fiscal expenditure responsibility and administrative responsibility. They know the most about the needs of the public and are the most able to accomplish practical things for the public. Therefore, at the present stage, the local government should not only consider economic growth or GDP as criteria but also take action based on whether they can provide better public services instead of simply aiming at increasing revenue.

Second, the public should be encouraged to supervise and constrict the government’s behavior. Relatively speaking, the Chinese government mainly aimed at local economic development and the promotion of officials in previous years, while largely ignoring public expenditure that would benefit the people’s welfare, such as environmental protection. The government invested more fiscal funds into construction, which can enhance economic revenue in a short time, and took the path of “pollution first, governance later”, similar to most industrialized countries, which is harmful to environmental protection and a harmonious coexistence between humans and nature. Furthermore, against the background of “carbon peaking and carbon neutrality goals” at the present stage and the comprehensive transition of the economy and society to green and low-carbon development, the ideology should completely change. Public supervision should especially be used fully to constrict local governments’ behavior, thereby forcing local governments to change the fiscal expenditure structure, finally solving the conflict between the people’s growing demand for a better life and the unbalanced and inadequate development and thus achieving a harmonious coexistence between humans and nature.

## Figures and Tables

**Table 1:** Summary of the most important articles of the literature.

Types	Ref. Author(s), Year	Methodology	Sample (If It Is Empirical)	Main Conclusions	Ref. No.
Fiscal expenditure structure	Wu et al., (2013)	Fixed effect model	Data of 27 provinces in China from 2000 to 2009	The appeal for political achievements urges local officials to allocate limited resources to areas conducive to economic growth, thereby reducing financial expenditures in people’s welfare areas such as environmental protection, which is harmful to efforts to improve environmental quality.	[14]
	Halkos and Paizanos (2013)	Fixed effect model; Two-stage least squares (2SLS); GMM	A panel of 77 countries for the time period 1980–2000.	Fiscal expenditure in favor of economic growth is bad for environment quality improvement.	[15]
	He et al., (2018)	Fixed effects; 2SLS	seven seriously polluted cities in China, from the period 2007–2015	The expenditure structure in favor of public services, such as environmental protection, and education, is conducive to reducing environmental pollution.	[16]
	Halkos and Paizanos (2017)	Dynamic Fixed Effects	panel data for 94 countries for the period 1970–2008	There is a significant alleviating direct effect of government expenditure on SO_2_ and NO_x_ emissions, which increases with the level of economic growth and democracy.	[17]
	Chen and Lu (2014)	Fixed Effects	Panel data of 112 prefecture -level cities in China from 2007 to 2009	Increasing the share of non-economic fiscal expenditure is conducive to alleviating production-generated pollution.	[18]
	Yu and Yang (2016)	Fixed Effects	Panel data of 287 cities in China from 2007 to 2013	Non-economic public expenditure would promote the technological innovation of emission reduction and pollution remediation, and thus the environmental quality could be improved at the same time as reducing pollutant emissions.	[19]
	Lu et al., (2015)	Fixed Effects; GMM	Data of 103 key environmental protection cities in China from 2007 to 2012	Increasing the share of China’s non-economic fiscal expenditure is conducive to alleviating the regional emission levels of consumption-generated pollutants.	[20]
Vertical fiscal imbalance	Bordignon et al., (2013)	Theoretical model; Dynamic Fixed Effects	Data of 89 chief provincial towns in Italy from 1988 to 1997	Under the fiscal decentralization system, the central government has more revenue and fewer expenditures, whereas local governments have less revenue and more expenditures, and the vertical fiscal imbalance arises at the historic moment.	[21]
	Boadway R. (2004)	Theoretical model	n.a.	Vertical fiscal imbalance is a common phenomenon in countries with decentralized systems.	[22]
	Jiménez-Rubio, D. (2011a)	Fixed Effects	A panel data of the highly decentralized Canadian provinces during the period 1979 to 1995.	Vertical fiscal imbalance is a common phenomenon in all decentralized countries, and moderate vertical imbalance is beneficial.	[23]
	Jiménez-Rubio, D. (2011b)	Fixed Effects	a panel of 20 OECD countries over a thirty-year period (1970–2001)	There are vertical imbalances in countries with decentralized systems. As long as they are maintained within an appropriate range, it will promote the optimization of local public expenditure structure.	[24]
	Bardhan and Mookherjee (2003)	Theoretical model	n.a.	Excessive vertical fiscal imbalance will produce many negative effects and harm, such as distorting the public expenditure structure of local governments.	[25]
	Smith and Revell (2016)	A case study approach	Data from 1990 to 2010 for six cities in Argentina and Mexico	After analyzed the political decentralization and fiscal decentralization of six countries in Latin America, including Argentina and Mexico, and found that excessive power of the provinces would cause severe vertical fiscal imbalance.	[26]
	Chu et al., (2017)	Panel threshold model	China’s budgetary and extra budgetary revenue and expenditure data from 1994 to 2015	China’s decentralization system, with political centralization and economic decentralization, leads to more serious vertical fiscal imbalance than that in western countries.	[27]
	Duan and Zhan (2011)	Fixed Effects	Data of 114 districts and counties in Shanxi Province, China from 1994 to 2005	Fiscal transfer payments among governments are an important measure for easing vertical fiscal imbalance.	[28]
	Boetti et al., (2012)	OLS regressions; SFA model; DEA model	Data for 262 cities in Turin, Italy	Italian government’s enhancement of the degree of self-financing of lower-level governments was conducive to easing vertical fiscal imbalance.	[29]
	Amusa et al., (2008)	Ordinary Least Squares Regression (OLS)	Data from 237 local government (category A and B municipalities) in South Africa in fiscal year 2005/06	Enhancing the degree of local self-sufficiency was more beneficial to easing vertical fiscal imbalance, instead of fiscal transfer payments among governments.	[30]
	Oates, W. (1993)	n.a.	n.a.	The excessive vertical fiscal imbalance may make local governments rely too heavily on transfer payments. Local governments’ efforts would be decreased under the soft constraint, thereby damaging the financial autonomy of the local government.	[31]
	Chu and Shao (2018)	Dynamic panel data Model; GMM	Data at the provincial level in China from 2007 to 2015	The vertical fiscal imbalance affects the public expenditure behavior of the local government and causes distortion of the public expenditure structure.	[32]
	Jia et al., (2016)	Dynamic panel data Model; Fixed Effects	Chinese prefectural panel data set from 2001 to 2007	The vertical fiscal imbalance exacerbates the behavior of land finance, during which political promotion intensifies the behavior, which affects the sustainable development of China’s economy and society.	[33]
	Wang and Zhang (2017)	Fixed Effects; Instrumental Variable (IV) Regression	Using a large, unique county-level panel dataset for China from 1998 to 2006	When faced with vertical fiscal imbalance constraints, especially a high level of imbalance, local governments depend on tax revenue from polluting enterprises to ease pressure, which inevitably causes environmental pollution.	[34]
	Xi, P.H. (2017)	Fixed Effects	Sample panel data of China’s cities from 2003 to 2009	when faced with vertical fiscal imbalance, the fiscal expenditure may be biased toward the industries which can bring more fiscal revenue, such as construction industry, real estate industry, etc., but these industries also cause environmental pollution.	[35]
	Xi et al., (2017)	Fixed Effects	China’s provincial panel data from 2003 to 2011	Under the influence of vertical fiscal imbalance, with respect to the fiscal expenditure structure, governments may lean more towards “resource-intensive” projects, represented by infrastructure construction. Such expenditure increases the GDP in a short time but causes irreversible pollution.	[36]
	Devarajan et al., (1996)	Theoretical model; Fixed effects	Data for 43 developing countries from 1970–1990	The sum of expenditures, including general public service, public safety, science and technology, social security, education, culture, sports, and media, as well as energy conservation and environmental protection, was viewed as expenditure on people’s welfare.	[37]
	Li et al., (2021)	Fixed effects; Pooled Effects; Random effects	Data of Pakistan from 2000 to 2018	The vertical fiscal imbalance causes environmental degradation by changing the industry structure, and also restrains environment supervision.	[38]
	Huang and Zhou (2020)	Fixed effects; Quantile Regression	The panel data of China’s 30 provincial level from 1999 to 2016	In China, a higher level of vertical fiscal imbalance causes even worse environmental pollution.	[39]

n.a. means not applicable.

**Table 2:** Classification and descriptions of variables.

Variable Type	Variables	Sign	Description
Explained Variables	Per capita wastewater	pww	Discharge amount of wastewater (10,000 tons) per capita
	Per capita waste gas	pwg	Discharge amount of waste gas (billion cubic meters) per capita
	Per capita solid waste	psw	Discharge of solid waste (10,000 tons) per capita
Core Explanatory Variables	Vertical fiscal imbalance	vfi	(1 − Fiscal revenue decentralization/Fiscal Expenditure Decentralization (1 — Local government’s gap ratio of fiscal self-sufficiency)) × 100 (%)
	Fiscal expenditure structure	exp	Expenditure on people’s welfare/Total fiscal expenditure × 100 (%)
Control Variables	Foreign direct investment	fdi	Amount of foreign capital actually used in the region (100 million CNY)
	Energy consumption structure	ecs	Proportion of coal consumption in total energy consumption (%)
	Industrial structure	is	Proportion of secondary industry, i.e., the industrial added value, in GDP (%)
	Urbanization rate	urba	Proportion of urban population in total provincial population (%)
	Real GDP per capita	rgdp	Real GDP per capita (CNY)
	Population density	pd	Proportion of total population in the area of this region (per capita square kilometer)
	Fixed asset investment	fai	Proportion of fixed asset investment in GDP (%)
	Built-up area	bua	Built-up area (square kilometer)

**Table 3:** Descriptive statistics of main variables.

Variable	Obs	Mean	Std. Dev	Min	Max
**Explained variables**					
Per capita wastewater (lnpww)	403	2.501	0.619	0.127	3.874
Per capita waste gas (lnpwg)	403	1.266	0.858	−3.944	3.263
Per capita solid wastes (lnpsw)	403	0.463	0.948	−3.979	3.241
**Core Explanatory Variables**					
Expenditure structure (lnexp)	403	3.945	0.122	3.577	4.327
Vertical fiscal imbalance (lnvfi)	403	4.152	0.309	2.874	4.605
**Control Variables**					
Industrial structure (lnis)	403	3.831	0.226	2.949	4.235
Foreign direct investment (lnfdi)	403	3.276	1.711	−1.897	5.985
Economic development level (lnrgdp)	403	7.624	2.819	0.437	15.424
Energy consumption structure (lnecs)	403	2.993	0.502	0.198	4.486
Population density (lnpd)	403	5.317	1.497	0.858	8.264
Built-up area (lnbua)	403	6.984	0.862	4.367	8.853
Fixed asset investment (lnfai)	403	8.789	0.423	6.800	9.564
Urbanization rate (lnurba)	403	3.970	0.267	3.068	4.495

**Table 4:** Basic regression results of the effects of vertical fiscal imbalance on the pollution reduction effect of the expenditure structure (three types of waste per capita).

Variable	Wastewater per Capita		Waste Gas per Capita		Solid Waste per Capita	
	(1)	(2)	(3)	(4)	(5)	(6)
lnexp	3.571	−6.334 **	−12.451 ***	−10.432 ***	−11.824 ***	−16.132 ***
	(0.98)	(−2.18)	(−5.35)	(−3.85)	(−6.85)	(−7.34)
lnvfi		25.793 ***		0.536		20.85 ***
		(3.01)		(0.11)		(6.20)
lnexp × lnvfi		3.837 ***		0.632		3.327 ***
		(6.47)		(0.19)		(6.28)
lnrgdp	0.315 ***	0.422 ***	−0.076 *	−0.085 *	−0.218 ***	−0.087 **
	(4.16)	(4.32)	(−1.83)	(−1.88)	(−3.56)	(−2.37)
lnis	6.852 **	6.735 **	1.425	1.425	4.105 **	3.012
	(2.32)	(2.49)	(0.86)	(0.85)	(2.32)	(1.38)
lnurba	3.724	1.257	3.456	3.231	0.745	−0.729
	(0.85)	(0.35)	(0.76)	(0.72)	(0.45)	(−0.56)
lnecs	1.732	0.534	0.897	0.872	5.531 ***	4.597 ***
	(0.78)	(0.28)	(0.46)	(0.67)	(4.13)	(2.87)
lnpd	−13.87	4.891	−2.137	−1.715	−4.165	9.435 **
	(−1.56)	(0.56)	(−0.57)	(−0.38)	(−1.08)	(2.25)
lnfdi	1.573 **	1.367 **	−0.189	0.013	−2.736 ***	−2.571 ***
	(2.32)	(2.38)	(−0.14)	(0.13)	(−7.45)	(−8.35)
lnbua	−7.154 **	−8.107 **	−2.514	−1.738	−2.470	−2.127
	(−2.19)	(−2.26)	(−0.83)	(−0.80)	(−1.25)	(−0.90)
lnfai	−2.653 **	−3.098 ***	−0.089	0.113	0.876	0.610
	(−2.35)	(−2.87)	(−0.04)	(0.09)	(1.28)	(1.06)
cons	155.9 ***	89.39 *	7.335	3.351	−30.35	−89.45 ***
	(3.83)	(1.80)	(0.36)	(0.12)	(−1.45)	(−4.18)
Region-fixed effect	YES	YES	YES	YES	YES	YES
Time-fixed effect	YES	YES	YES	YES	YES	YES
N	403	403	403	403	403	403
R2	0.322	0.305	0.289	0.265	0.460	0.513

Note: ***, **, and * denote significance levels of 1%, 5%, and 10%, respectively; the numbers in the brackets are the statistical values.

**Table 5:** Regression results of GMM.

Variable	Wastewater per Capita		Waste Gas per Capita		Solid Waste per Capita	
	(1)	(2)	(3)	(4)	(5)	(6)
L.lnpww	0.637 (0.73)	0.513 * (1.72)				
L.lnpwg			0.754 ** (2.35)	0.627 (0.09)		
L.lnpsw					0.684 ** (2.05)	0.526 ** (1.98)
lnexp	2.416	−4.125 *	−9.537 **	−5.267 *	−8.214 **	−12.125 ***
	(0.46)	(−1.65)	(−2.21)	(−1.87)	(−2.52)	(−5.34)
lnvfi		21.651 ***		0.472		18.43 **
		(2.74)		(0.09)		(2.25)
lnexp × lnvfi		2.165 ***		0.562		2.127 ***
		(2.97)		(0.12)		(5.08)
cons	129.352 ***	80.394 *	6.246	2.931	−28.531	−86.317 ***
	(2.95)	(1.69)	(0.26)	(0.11)	(−1.25)	(−3.18)
Control	YES	YES	YES	YES	YES	YES
Region-fixed effect	YES	YES	YES	YES	YES	YES
Time-fixed effect	YES	YES	YES	YES	YES	YES
N	372	372	372	372	372	372
AR(1)	0.019	0.038	0.022	0.037	0.025	0.034
AR(2)	0.246	0.774	0.053	0.064	0.846	0.758
Sargan	0.325	0.513	0.216	0.198	0.502	0.461

Note: ***, **, and * denote significance levels of 1%, 5%, and 10%; the numbers in the brackets are the statistical values. The GMM used was system GMM, and the command used was xtabond2.

**Table 6:** Regression results of pollution reduction effect of fiscal expenditure structure (total amount of three types of waste).

Variable	Total Amount of Industrial Wastewater		Total Amount of Industrial Waste Gas		Total Amount of Industrial Solid Waste	
	(1)	(2)	(3)	(4)	(5)	(6)
lnexp	−0.852 **	−0.864 **	−0.538	−0.641	−0.872 **	−2.975 ***
	(−2.18)	(−2.42)	(−0.90)	(−0.90)	(−2.12)	(−6.17)
lnvfi		0.578		0.154		5.372 ***
		(0.76)		(0.21)		(6.18)
lnexp × lnvfi		1.251		0.876		1.546 ***
		(1.18)		(0.56)		(3.38)
cons	4.654	8.753 *	−26.16 ***	−29.983 ***	−22.761 ***	−45.49 ***
	(1.23)	(1.89)	(−3.99)	(−3.92)	(−4.53)	(−8.79)
Control	YES	YES	YES	YES	YES	YES
Region-fixed effect	YES	YES	YES	YES	YES	YES
Time-fixed effect	YES	YES	YES	YES	YES	YES
N	403	403	403	403	403	403
R2	0.201	0.223	0.378	0.389	0.496	0.589

Note: ***, **, and * denote significance levels of 1%, 5%, and 10%, respectively; the numbers in the brackets are the statistical values.

**Table 7:** Regression results of pollution reduction effect of fiscal expenditure structure (pollution intensity).

Variable	Pollution Intensity (Wastewater)		Pollution Intensity (Waste Gas)		Pollution Intensity (Solid Waste)	
	(1)	(2)	(3)	(4)	(5)	(6)
lnexp	−1.243 ***	−0.997 ***	0.516	0.338	0.061	−2.006 ***
	(−5.15)	(−3.89)	(0.91)	(0.46)	(0.10)	(−4.92)
lnvfi		0.728		0.341		5.789 ***
		(1.12)		(0.31)		(6.89)
lnexp × lnvfi		1.129		1.910		0.30 ***
		(1.42)		(1.19)		(8.43)
cons	24.42 ***	26.13 ***	−14.65 **	−20.35 ***	−11.89 **	−37.13 ***
	(8.92)	(6.93)	(−2.17)	(−2.76)	(−2.18)	(−6.87)
Control	YES	YES	YES	YES	YES	YES
Region-fixed effect	YES	YES	YES	YES	YES	YES
Time-fixed effect	YES	YES	YES	YES	YES	YES
N	403	403	403	403	403	403
R2	0.797	0.799	0.090	0.109	0.098	0.356

Note: ***, ** denote significance levels of 1%, 5% respectively; the numbers in the brackets are the statistical values.

**Table 8:** Impact of fiscal pressure on pollution reduction effect of fiscal expenditure structure.

Variable	Wastewater per Capita		Waste Gas per Capita		Solid Waste per Capita	
	(1)	(2)	(3)	(4)	(5)	(6)
lnexp	2.563	−8.487 *	−11.45 ***	−13.42 ***	−11.31 ***	−18.97 ***
	(0.73)	(−1.93)	(−5.76)	(−4.87)	(−6.15)	(−7.89)
lnpre		12.97 ***		1.042		9.654 ***
		(4.18)		(0.47)		(6.89)
lnexp × lnpre		14.98 ***		1.932		12.86 ***
		(3.47)		(0.84)		(6.76)
cons	166.814 ***	125.357 **	6.652	−0.682	−29.57	−81.78 ***
	(3.89)	(2.47)	(0.35)	(−0.03)	(−1.41)	(−3.83)
Control	YES	YES	YES	YES	YES	YES
Region-fixed effect	YES	YES	YES	YES	YES	YES
Time-fixed effect	YES	YES	YES	YES	YES	YES
N	403	403	403	403	403	403
R2	0.298	0.343	0.265	0.278	0.465	0.572

Note: ***, **, and * denote significance levels of 1%, 5%, and 10%, respectively; the numbers in the brackets are the statistical values.

**Table 9:** Heterogeneity test.

Variable	Eastern			Central			Western		
	Wastewater per Capita	Waste Gas per Capita	Solid Waste per Capita	Wastewater per Capita	Waste Gas per Capita	Solid Waste per Capita	Wastewater per Capita	Waste Gas per Capita	Solid Waste per Capita
lnexp	−4.144 **	−10.145	−14.425	−6.447 **	−12.715 **	−17.395 *	−8.919 ***	−14.841 ***	−19.065 ***
	(−1.98)	(−1.46)	(−1.62)	(−2.38)	(−2.46)	(−1.79)	(−5.84)	(−5.20)	(−6.47)
lnvfi	23.886	5.983	18.702 *	25.261 ***	6.131 *	20.226 ***	27.422 ***	8.563 ***	22.600 ***
	(1.58)	(0.673)	(1.67)	(2.71)	(1.71)	(2.87)	(2.98)	(3.91)	(3.77)
lnexp×lnvfi	2.326 *	0.452	2.253 *	5.172 **	0.726 *	3.370 **	7.271 ***	0.899 **	5.367 ***
	(1.75)	(1.54)	(1.67)	(2.45)	(1.93)	(2.31)	(6.73)	(2.49)	(7.79)
cons	80.48	1.915 *	87.330	81.321 **	3.177 **	89.647 **	85.244 **	1.557 **	91.085 **
	(1.61)	(1.72)	(1.35)	(2.16)	(2.31)	(2.38)	(2.13)	(2.32)	(2.50)
Control	YES	YES	YES	YES	YES	YES	YES	YES	YES
Region-fixed effect	YES	YES	YES	YES	YES	YES	YES	YES	YES
Time-fixed effect	YES	YES	YES	YES	YES	YES	YES	YES	YES
N	143	143	143	104	104	104	156	156	156
R2	0.705	0.339	0.642	0.764	0.574	0.766	0.697	0.392	0.536

Note: ***, **, and * denote significance levels of 1%, 5%, and 10%, respectively; the numbers in the brackets are the statistical values.

## Data Availability

Not applicable.
